# Therapeutic Effects of Transcranial Magnetic Stimulation on Visuospatial Neglect Revealed With Event-Related Potentials

**DOI:** 10.3389/fneur.2021.799058

**Published:** 2022-01-24

**Authors:** Lin-lin Ye, Huan-xin Xie, Lei Cao, Wei-qun Song

**Affiliations:** Department of Rehabilitation, Xuanwu Hospital, Capital Medical University, Beijing, China

**Keywords:** cognition, posterior parietal cortex, rehabilitation, transcranial magnetic stimulation, visuospatial neglect, event-related potential, P300

## Abstract

This study aimed to investigate changes in attention processing after low-frequency repetitive transcranial magnetic stimulation (rTMS) over the left posterior parietal cortex to better understand its role in visuospatial neglect (VSN) rehabilitation. The current study included 10 subacute stroke patients with VSN consecutively recruited from the inpatient stroke rehabilitation center at Xuanwu Hospital (the teaching hospital affiliated with Capital Medical University) between March and November 2019. All patients performed a battery of tasks (including line bisection, line cancellation, and star cancellation tests) two weeks before treatment and at the beginning and end of treatment; the attentive components of the test results were analyzed. In addition, low-frequency rTMS was used to stimulate the left posterior parietal cortex for 14 days and event-related potential data were collected before and after the stimulation. Participants were evaluated using a target-cue paradigm and pencil-paper tests. No significant differences were detected on the battery of tasks before rTMS. However, we found that rTMS treatment significantly improved the response times and accuracy rates of patients with VSN. After rTMS, the treatment side (left) amplitude of P300 following an event-related potential was higher than that before treatment (left target, *p* = 0.002; right target, *p* = 0.047). Thus, our findings suggest that rTMS may be an effective treatment for VSN. The observed increase in event-related potential amplitude supports the hypothesized compensational role of the contralesional hemisphere in terms of residual performance. Our results provide electrophysiological evidence that may help determine the mechanisms mediating the therapeutic effects of rTMS.

## Introduction

Visuospatial neglect (VSN) is a neuropsychological disorder that impairs higher-level cognition, particularly spatial attention (SA). Deficits in SA not only impact the processing of sensory events but also affect global processing (1). Negative impacts on SA often occur after a stroke in the right hemisphere and manifest as a failure to respond to stimuli in the contralateral visual field (2). While spontaneous recovery from VSN can occur, nearly 40% of patients continue to have symptoms (3). Considering that VSN is a highly debilitating condition that seriously affects the patient recovery and quality of life (4), the development of novel therapeutic methods is needed.

According to the interhemispheric competition model, direct attention toward the contralateral space is competed by the parietal lobes with each other, resulting in a reciprocal interhemispheric inhibition. Damage to the right parietal cortex will lead to the disinhibition of the intact, left parietal cortex (5); however, reducing this imbalance is possible. Another hypothesis is also considered to be the key mechanism leading to neglect. The destruction of the functional connection between the attention networks of the two cerebral hemispheres. Functional magnetic resonance imaging (fMRI) studies have shown that VSN recovery is related to the recovery and rebalancing of activity between damaged and undamaged hemispheres, especially in the parietal cortex (6).

Transcranial magnetic stimulation (TMS) is considered to be a promising treatment for VSN. Based on the interhemispheric rivalry model (5), repetitive TMS (rTMS) inhibits neural networks associated with attention in the intact hemisphere, which can normalize interhemispheric cortical excitability and ameliorate the symptoms of VSN. Emerging evidence suggests that rTMS might be effective for improving the behavioral deficits induced by VSN (7), and functional imaging provides some evidence for changes in the attentional network following TMS. Studies using low-frequency TMS have shown that visuospatial attention is impaired by disruption of the right posterior parietal cortex (8). However, this evidence does not consider interindividual variability or attentional processing speed. The effects of TMS on visuospatial attention processing and cognition function in VSN patients are still poorly understood. To understand these roles, a high temporal resolution approach is required to capture the dynamics of corticocortical interaction and to identify the effects of TMS on the different stages of visual attention processing.

An event-related potential (ERP) is an electrophysiological measure of the cortical networks involved in cognitive processes, such as attention and working memory (9). Multiple studies have shown that P300, a positive component of ERPs that peaks at ≥300 ms, responds to the sum of activities of multiple generators located in a wide range of cortical and subcortical areas (10). The cognitive component of an ERP reflects changes in attentional resources as well as environment-related attentional updates regulated by attention (11). In fact, many diseases of the neurologic and psychiatric systems, including schizophrenia, migraine, and depression, reduce P300 amplitude or increase its peak latency, indicating a deficit in cognitive processing. A previous study on healthy individuals showed that TMS stimulus led to an increase in P300 amplitude on the stimulation side in an ERP (12). However, to our knowledge, few studies have assessed the effects of TMS treatment using a visual paradigm.

Our previous assessment of patients with VSN revealed that changes in the visual paradigm were a late (rather than early) component of ERPs (13). Based on this, we sought to evaluate ERPs in patients before and after TMS. We hypothesized that TMS would have an impact on VSN and that this would be reflected through P300. Therefore, we aimed to observe the electrophysiological changes of attention processing in patients with VSN before and after TMS treatment. Additionally, we expected improvements in clinical behavioral evaluation outcomes.

## Materials and Methods

### Study Design

**Figure 1:**
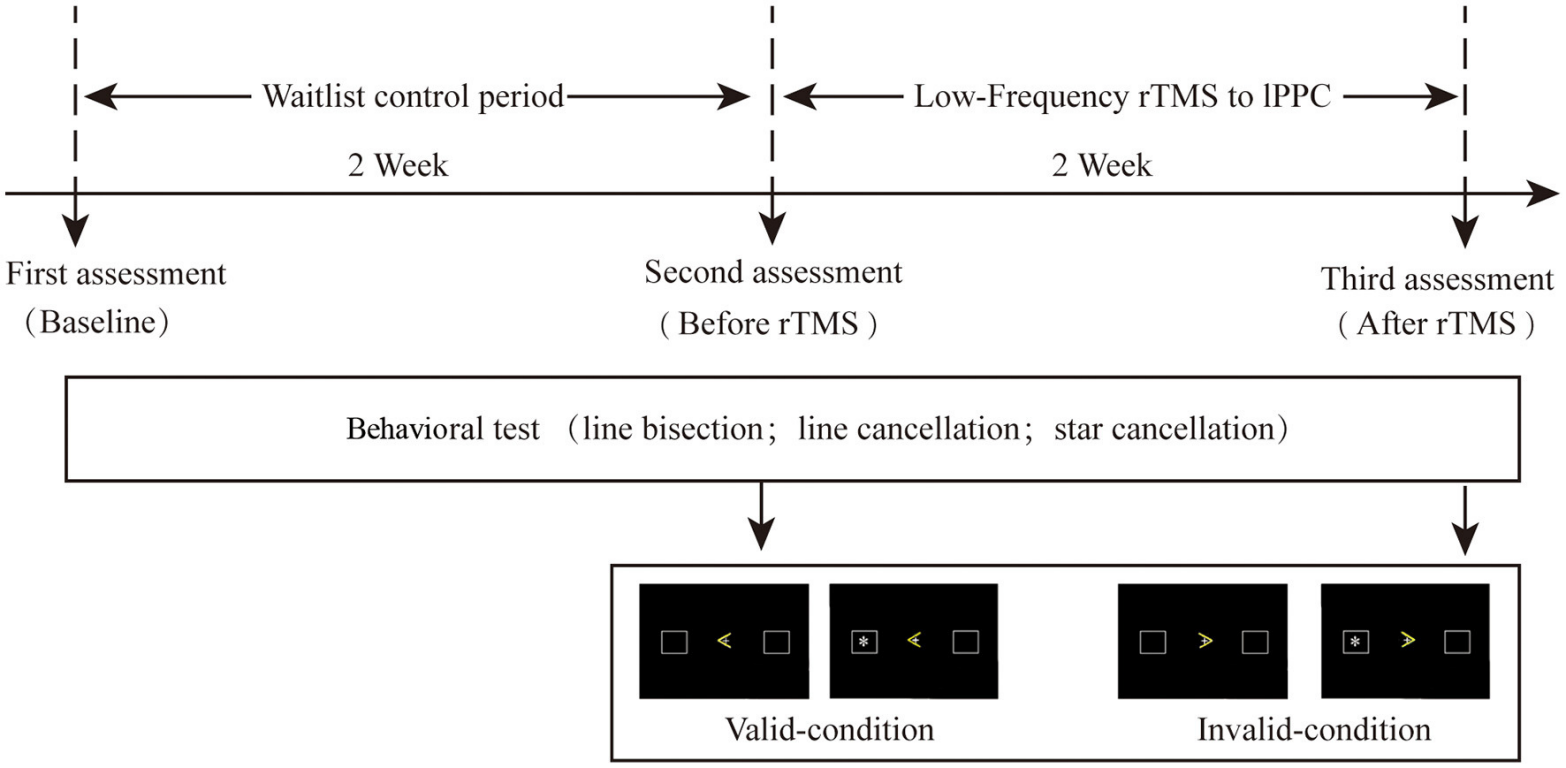
Event sequence of the experimental design; cues: “>” and “<”; target: “*”.

The study duration was four weeks (Figure 1), comprising a waiting period of two weeks (i.e., continuing treatment as usual) followed by two weeks of rTMS therapy at four sessions per day. The first behavioral assessment was conducted at the beginning of the waiting period. All patients received physical therapy (PT). ERP and behavior were retested on the first day as well as after two weeks of TMS. A 30-min PT program was applied immediately after stimulation, mainly focusing on upper and lower limb rehabilitation. This study was approved by the Ethics Committee of Xuanwu Hospital (the teaching hospital affiliated with Capital Medical University; approval number [2019]016) and was conducted in accordance with the principles of the Declaration of Helsinki and its later amendments. All patients provided written informed consent before their participation.

### Participants

Ten participants were consecutively recruited from the inpatient stroke rehabilitation clinic of the Department of Rehabilitation at Xuanwu Hospital (the teaching hospital affiliated with Capital Medical University) between March and November 2019. The inclusion criteria were as follows: (1) age 18–80 years; (2) the presence of a right brain stroke (cerebral infarction or hemorrhage) confirmed by computed tomography (CT) or magnetic resonance imaging (MRI), with a clinical course of at least four weeks; (3) right handedness; (4) VSN according to a line bisection test, a star cancellation test, or a clinical examination; and (5) the provision of informed consent by the patient and their family.

The exclusion criteria were as follows: (1) the presence of new-onset infarction, hemorrhage lesions, or other worsening conditions; (2) the presence of severe uncorrectable visual impairment and/or visual field disturbance; (3) the presence of hemianopsia (diagnosed with perimetry); (4) a previous history of claustrophobia; (5) an epilepsy diagnosis; (6) the presence of metal implants; (7) a Mini-Mental State Examination score <17; (8) being uncooperative during examination; and (9) having used tricyclic antidepressants drugs at any time within the six months before enrollment.

### Resting Motor Threshold

A Magstim Rapid2 device (Magstim, Sheffield, UK) with a 70 mm figure-eight coil was used for conducting this measurement. In all participants, the left hemisphere motor threshold was determined for the minimum intensity of a single-pulse TMS (>50 V), or if no visible motor evoked potentials (MEPs) were detected in the first interosseous dorsal muscle on at least 5 of the 10 consecutive trials in the primary motor cortex. The two electromyographic (EMG) recording electrodes were placed >2 cm apart. EMG responses were recorded with a Necolet VikingQuest monitor (VIASYS Healthcare, Inc., Wisconsin, USA).

### rTMS Protocol

The same magnetic stimulator was used for this component of the study. We chose an offline low-frequency TMS protocol (1-Hz, 7.5-min, figure-eight coil) to suppress cortical excitability. The stimulation frequency was set at 1.0 Hz (equivalent to 90% of the resting motor threshold [RMT]), with a total of 450 pulses per session (including two trains with 225 pulses each). There was <1 min between the train intervals. A locating cap was used to orient to the posterior parietal cortex (PPC), corresponding to P3 with regard to the 10–20 system of electrode placement. The coil was placed tangentially to the scalp and positioned at 45° to the midsagittal axis at the left PPC; the coil was fixed with a metal clamp. Patients were asked to sit quietly, close their eyes, and keep their head still. The rTMS was administered twice a day daily for two weeks. The time interval between the two sessions was 12 h.

### Clinical Behavioral Tasks

#### Line Bisection Task

On a 295 × 210-mm A4 paper, five parallel line segments were equidistantly distributed, with lengths of 16, 14, 12, 10, and 8 cm. Patients were instructed to mark the midpoint of each line segment. The distance between the marked and actual midpoints was measured as R. The length of the line segment was denoted by L. The neglect degree was expressed by the following formula: [R/(L/2)] × 100%; >12% indicated VSN.

#### Line Cancellation Task

Thirty randomly selected black line segments (15–20 mm in length and 1 mm in width) were placed on the left and right quadrants of a 295 × 210-mm A4 paper with 15 lines in each direction. The patient was required to mark all visible line segments. VSN was indicated if more than three line segments were crossed out on the left as compared to the right.

#### Star Cancellation Task

In this task, scattered stars, small stars, letters, and words were symmetrically displayed on a 295 × 210-mm A4 paper. The patient was requested to mark all the small stars (27 on the left, 27 on the right, and 2 in the middle) on the test paper. When the left omission was ≥5, the patient was considered to have VSN.

The behavioral results were evaluated by two neurologists who were blinded to the treatment.

### ERP Assessment and Procedure

Stimulation was presented by E-Prime 4.5 software (Psychology Software Tools, USA). The participants sat 50 cm away from the 14-inch screen, facing the center of the screen. Responses were given with the left and right mouse button of the laptop computer (cues: “>” and “<”; target: “^*^”). The cues were located at the center of the screen, and the target stimuli were presented in two 15-mm squares placed 60 mm left and 60 mm right, respectively, from the center of the screen. The ERP task comprised 16 sessions, each of which had 40 trials. Each trial started with a fixed cross in the center. The background was presented for 800–1000 ms, and the targets were preceded by a cue delivered 1,400–1,800 ms before the target onset; the target appeared for 100 ms on either the left or right side of the screen (with equal probability). Participants were asked to press the left or right button as soon as possible to detect the appearance of the target on the same side. The maximum response time was 1,200 ms. After the button was pressed, the screen was cleared, and the next trial began in 1,000 ms. All participants completed 640 trials. Conditions in which the cue correctly indicated the location of the target were recorded as “valid,” and conditions in which the cue pointed to the contralateral side of the target were recorded as “invalid.” The valid-to-invalid ratio was 80:20. Before completing the test, participants were informed that both accuracy and response times were equally important. During the testing period, participants were allowed to rest for 1–2 min between sessions, if desired.

### ERP Recording

The ERP was recorded using a Neuroscan system with 64 electrodes placed on the scalp in an EEG cap, according to the international 10–20 system. (Compumedics USA Inc., Charlotte, NC, USA). The reference electrodes were placed on the bilateral mastoids and both links and eye movements were monitored through electrodes placed on the outer canthi of the left and right eyes as well as above and below the left eye. EEG data were sampled at 250 Hz and filtered using a 0.05–80 Hz filter. Impedances were maintained at <5 K Ω. Electrooculogram correction was performed via blink filtering, and visually detectable artifacts were removed before signal averaging. The data over ±100 μ V were automatically rejected as artifacts. The data were initially segmented into 1,000-ms epochs (200-ms pre, 800-ms post). Only the trials for which correct responses were available were analyzed.

### ERP Analysis

The analysis of the P300 components included the presence of waveforms, latency, and amplitude. The average P300 components were obtained at the F3, F4, C3, C4, P3, and P4 electrode sites. The P300 latency was identified manually in the time window of 300–700 ms and amplitude was defined as the maximum peak within the same time window.

### Statistical Analyses

Data analysis was performed using SPSS version 22.0 (IBM, Armonk, NY). Repeated-measures analysis of variance (ANOVA) was used to compare data from the pre- and post-treatment stages in all patient groups, and *P* values were corrected using Greenhouse–Geisser correction. ERP data were examined using three-way repeated measures ANOVA (target × hemisphere × recording site) to evaluate the main effects of sessions. The assumption of sphericity was tested using Mauchly's test, and adjustments were applied using the Greenhouse–Geisser correction. Pair-wise comparisons were performed for pre- and post-rTMS and were subsequently Bonferroni-corrected. The statistical significance level was set at *p* < 0.05.

## Results

### Patient Characteristics

A total of 12 patients were initially included in the current study; however, one patient failed to complete the ERP evaluation due to fatigue and one patient was excluded due to artifacts. Therefore, 10 patients were included in the final analysis (nine men and one woman). General patient demographics and data from the battery of tasks administered in the current study are summarized in Table 1. The average participant age was 57.90 ± 11.93 years. The average participant course was 68.60 ± 43.95 days.

**Table 1:** Demographic and general clinical patient data.

**Patient**	**Sex**	**Age (years)**	**Duration after stroke (days)**	**Stroke type**	**Lesion site**	**Line bisection (deviation %)**			**Line cancellation (all omission)**			**Star cancellation (all omission)**		
						**Baseline**	**Before rTMS**	**After rTMS**	**Baseline**	**Before rTMS**	**After rTMS**	**Baseline**	**Before rTMS**	**After rTMS**
1	M	65	149	CI	T CS BG	56.07	49	17.6	15	15	5	41	38	14
2	M	62	31	CH	T P BG	69.96	53.66	7	20	17	0	48	39	2
3	M	49	40	CI	F T BG	33	14	6.4	9	5	0	36	26	1
4	M	73	31	CI	F P BG	25.4	17.35	7.6	9	5	0	39	29	2
5	M	65	64	CI	T F P BG CS	68.37	50	14.58	27	23	9	50	46	25
6	F	30	59	CH	F	38.26	33	17.5	15	15	4	48	40	11
7	M	64	50	CI	BG CS T	62.96	49	22	12	10	3	30	28	11
8	M	54	148	CI	BG CS	28.7	20.2	11.1	4	4	1	25	21	7
9	F	62	68	CI	T F P BG CS	75.23	55	8	13	9	0	37	25	0
10	M	55	46	CH	F T P CS	42.27	38	10	5	3	0	13	9	2

*M, male; F, female; CH, cerebral hemorrhagic; CI, cerebral ischemic; CS: centrum semiovale; BG: basal ganglia; F, frontal lobe; p, parietal lobe; rTMS, repetitive transcranial magnetic stimulation, T, temporal lobe*.

### Adverse Events

All patients tolerated the intervention well without any adverse events, including mild events such as a slight headache.

### Behavioral Scores

**Figure 2:**
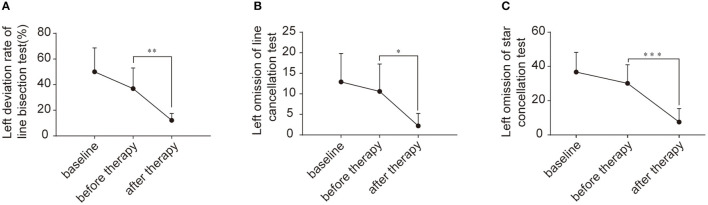
Behavioral scores for the enrolled patients. **(A)** Left deviation rate of line bisection test (%) between two weeks before treatment, beginning of treatment, and end of treatment. **(B)** Left omission of line cancellation test between two weeks before treatment, beginning of treatment, and end of treatment. **(C)** Left omission of star cancellation test between two weeks before treatment, beginning of treatment, and end of treatment. *p < 0.05; **p < 0.001; ***p < 0.0001.

VSN patients were assessed at three time points (two weeks before treatment, at the beginning of treatment, and at the end of treatment) using the paper-pencil test. The results are shown in Figure 2. Using a repeated measures ANOVA with Bonferroni correction, we found no significant difference in behavioral scores between baseline and before TMS in the line bisection test (F_(2, 27)_ = 17.410, *p* = 0.163), although there was a significant improvement across rTMS (F_(2, 27)_ = 17.410, *p* = 0.002). Likewise, there was no significant difference in the behavioral scores between baseline and before TMS in the line cancellation test (F_(2, 27)_ = 9.362, *p* = 1.000), although there was a significant improvement across rTMS (F_(2, 27)_ = 9.362, *p* = 0.01). Additionally, there were no significant differences in behavioral scores between baseline and before TMS in the star cancellation test (F_(2, 27)_ = 22.360, *p* = 0.483), although there was a significant improvement across rTMS (F_(2, 27)_ = 22.360, *p* < 0.001).

### Behavioral Analyses of Response Time and Accuracy Rate

**Figure 3:**
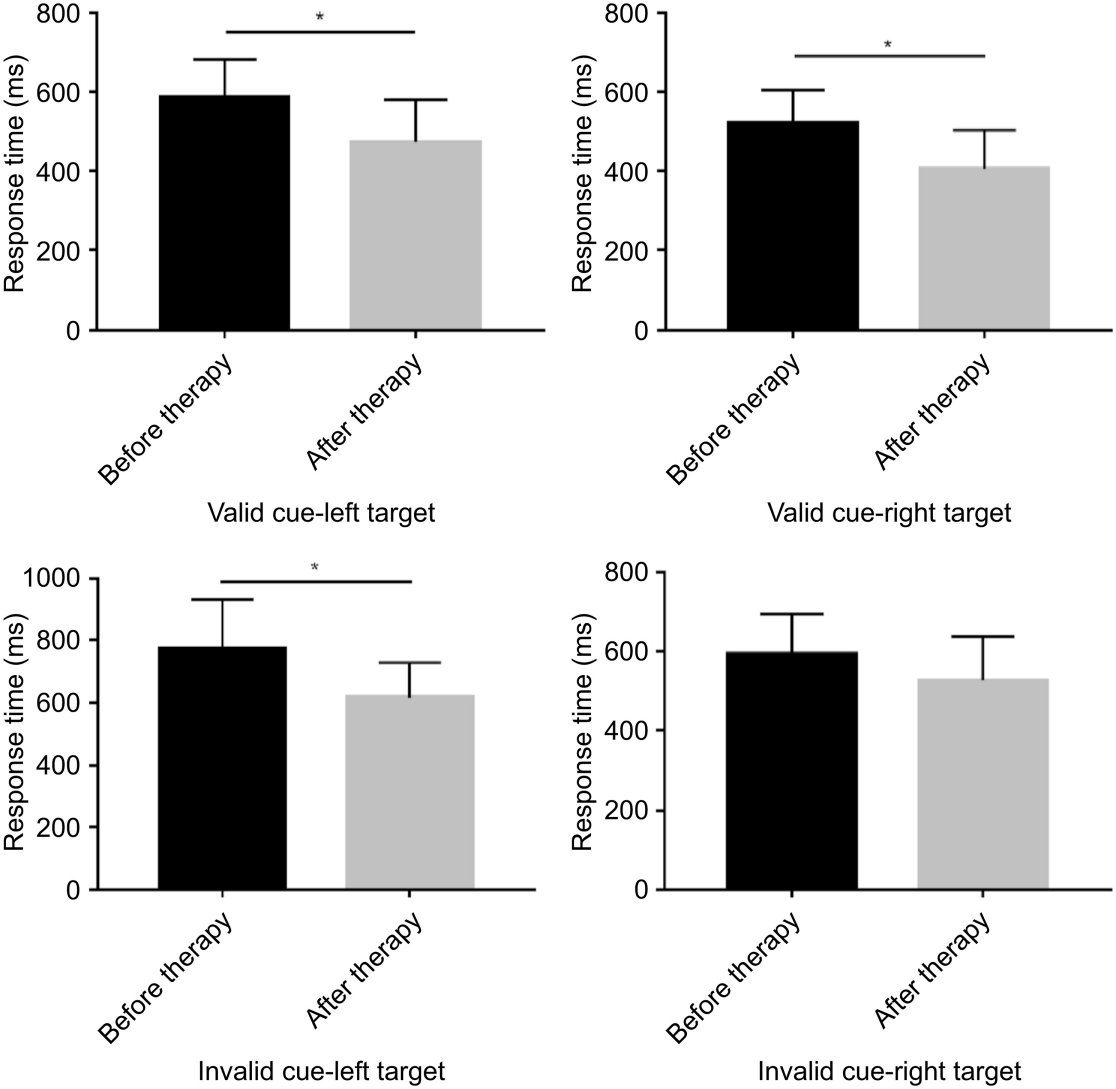
Bar graphs depicting the response time before and after therapy in the context of a valid or an invalid target, as well as a left-cue or right-cue target. *p < 0.05.

The behavioral analyses of the response time (RT) and accuracy rate under different contexts are summarized in Figure 2. The RT was comparable before and after rTMS in the VSN patients, although there was a significant difference after treatment (F_(1, 18)_ = 6.225, *p* = 0.023) under the valid cue and left target conditions, which showed a shorter RT after rTMS (472.12 ± 107.56 ms) as compared to before rTMS (585.70 ± 95.69 ms). Similarly, the analysis demonstrated a significant difference after treatment (F_(1,18)_ = 7.767, *p* = 0.012). The RT under the valid cue and right target condition showed that the response times before and after treatment were 519.80 ± 84.51 ms and 404.95 ± 99.20 ms, respectively. The RT under the invalid cue in the left target condition showed that the response times before and after treatment were 773.06 ± 157.35 ms and 617.75 ± 110.30 ms, respectively. The analysis also revealed a significant difference after treatment (F_(1,18)_ = 6.532, *p* = 0.020) within the right target condition, whereas the response times before and after treatment were 592.84 ± 102.04 ms and 527.25 ± 111.57 ms, respectively. However, there was no significant difference observed after treatment (F_(1,18)_ = 1.076, *p* = 0.313; Figure 3).

**Figure 4:**
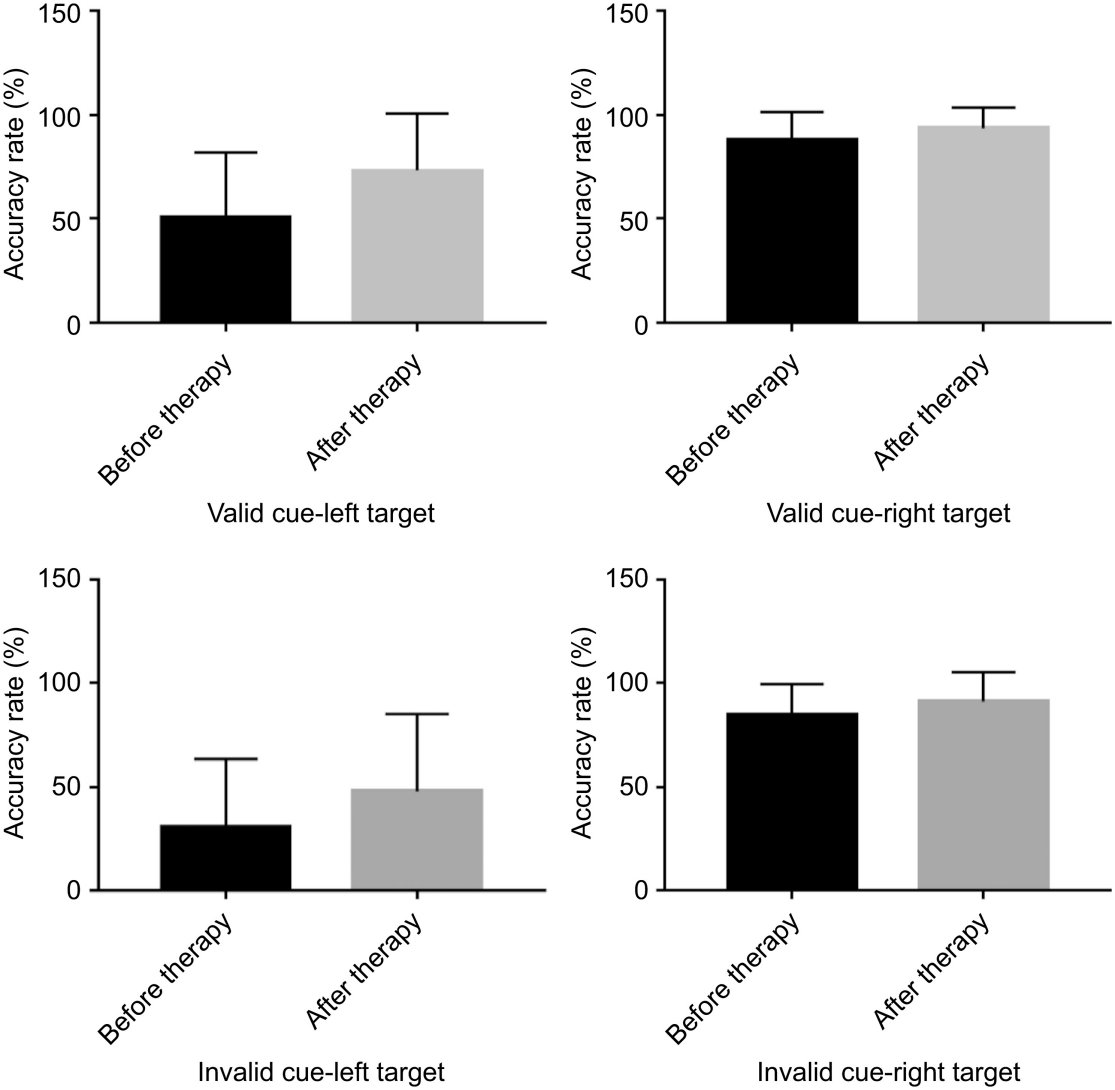
Bar graphs depicting the accuracy rate before and after therapy in the context of a valid or an invalid target, as well as a left-cue or right-cue target.

Regarding the accuracy rate, there were no significant differences before and after TMS between the left and right target conditions, regardless of whether the cue was valid or invalid (valid cue: left-target [*p* = 0.106] vs. right target [*p* = 0.298]; invalid cue: left-target [*p* = 0.278] vs. right target [*p* = 0.313]) (Figure 4).

### Electrophysiological Analyses of P300 Components

#### P300 Amplitude

**Figure 5:**
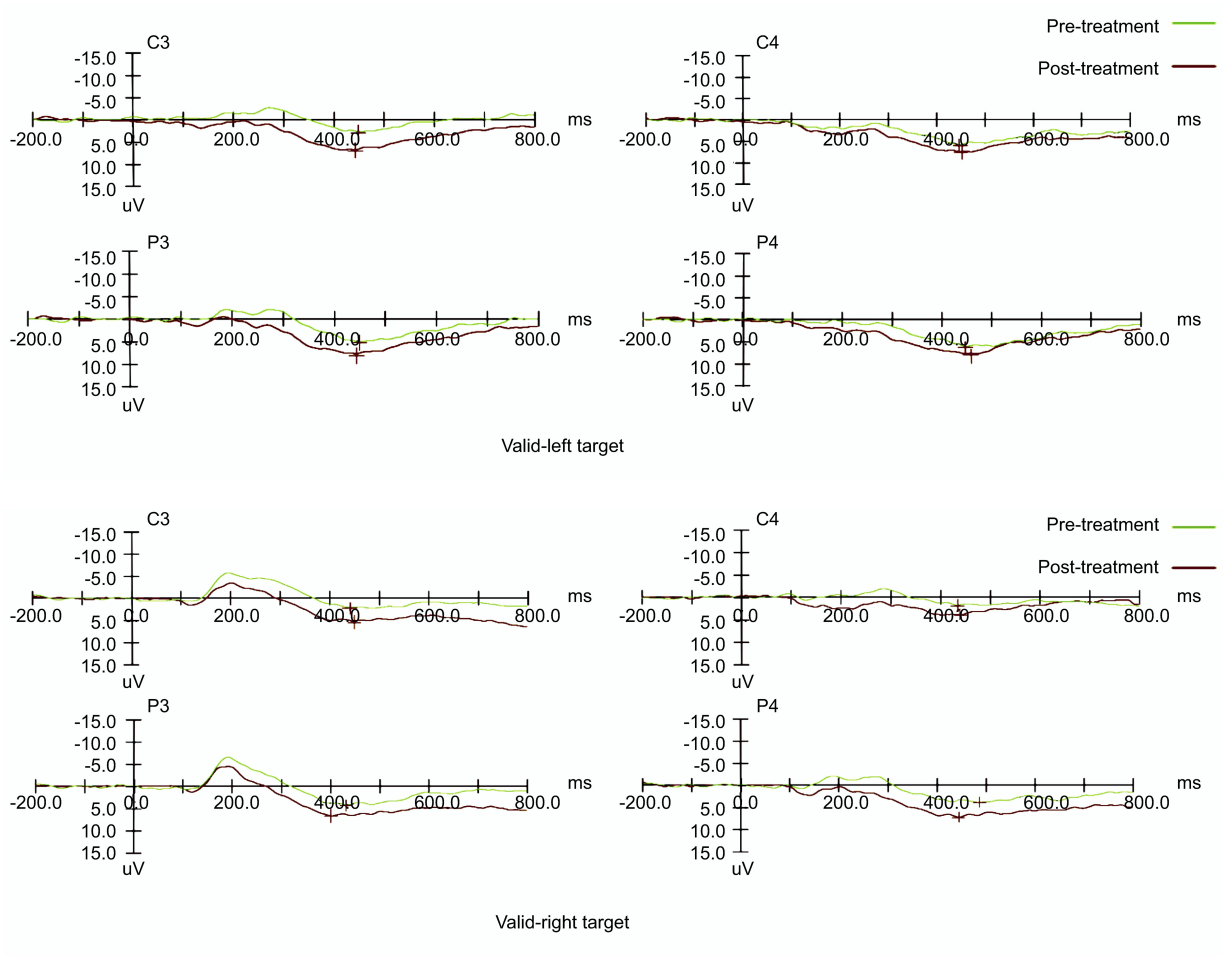
Event-related potential patterns effectively indicating P300 composition recorded at the P3 & P4 and C3 & C4 electrodes on the left and right sides before and after treatment.

Table 2 displays the mean amplitude and latency of P300. For P300 components, the signals from the reference electrode were converted to the mean of signals from the bilateral mastoid processes. The amplitude of the maximum crest in the time window was defined as the amplitude of specific ERP components, and the interval between the maximum crest and baseline was defined as the latency. Figure 5 shows the visual ERP grand averages in each group. For P300, we observed a higher mean P300 amplitude evoked over the contralateral visual target. There was a significant effect on amplitude in the left hemisphere (the treatment hemisphere), while using the left target (F_(1, 18)_ = 13.434, *p* = 0.002) and while using the right target (F_(1,18)_ = 4.539, *p* = 0.047). When the left and right hemispheres were compared, we observed no significant difference between the left and right targets (left hemisphere [*p* = 0.664], right hemisphere [*p* = 0.224]). No other main effects or interactions were significant in the current study (all *p* > 0.05).

**Table 2:** Amplitude and latency of the P300 component.

	**Amplitude (μV)**				**Latency (ms)**			
	**Left Target**		**Right Target**		**Left Target**		**Right Target**	
	**Left**	**Right**	**Left**	**Right**	**Left**	**Right**	**Left**	**Right**
	**hemisphere**	**hemisphere**	**hemisphere**	**hemisphere**	**hemisphere**	**hemisphere**	**hemisphere**	**hemisphere**
Pre-therapy	5.12 ± 3.22	7.75 ± 3.80	4.37 ± 4.56	5.82 ± 5.38	444.27 ± 71.86	449.27 ± 90.03	466.30 ± 68.14	507.90 ± 107.11
Post therapy	8.54 ± 3.67	8.46 ± 3.70	8.46 ± 5.22	8.56 ± 4.73	422.30 ± 63.48	446.27 ± 86.07	491.77 ± 129.08	478.53 ± 121.45

#### P300 Latency

The patients enrolled in this study showed a single peak P300 in the visual paradigm. There were no significant differences in latency before and after treatment (F_(1, 18)_ = 0.099, *p* = 0.757), with no evidence of interaction.

## Discussion

The results of the current study showed that interference with rTMS over the unaffected hemisphere can induce an improvement in VSN accompanied by a higher visual P300 amplitude. Therefore, cognitive compensation in the unaffected hemisphere may play a key role in improving VSN. We found that the performance of VSN patients taking the paper-pencil test was significantly improved after rTMS as compared with spontaneous recovery. This electrophysiological evaluation provides direct evidence of attention processes via a measure of brain activity.

The proportion of missed left targets in the ERP experiment was considerable, which is consistent with previously published reports (14). We found that participants also missed some of the right targets, but much less often than they missed the left targets. The paper-pencil test was conducted two weeks before rTMS, immediately before rTMS, and at the end of the last rTMS session. Interestingly, performance on this test was not significantly different before rTMS; however, after two weeks of rTMS treatment, there was a dramatic improvement in the paper-pencil test results for VSN patients. The deficit in lateralized attention is strong in the early stages of stroke. While this deficit can spontaneously recover to a limited extent over time, a much greater degree of improvement can be achieved through rTMS. At the behavioral level, rTMS improved the symptoms of VSN. However, as this effect could not be attributed to spontaneous recovery, we believe that rTMS has a positive effect on VSN.

It was a reasonable decision to select PPC as a site for rTMS stimulation. The PPC is a critical component of attention networks; intact PPC function was essential during the encoding, consolidation, and retrieval of an associability memory enhanced by surprising omissions (15). In a previous study, direct electrical stimulation was performed in seven patients with hemispheric gliomas during surgery with asleep/awake anesthesia, Stimulation of the superior parietal lobule caused a marked rightward deviation in all of the six patients with right hemisphere lesions (16). Thus, the PPC has been proposed to be a crucial node among cortical areas included in the network. Some fMRI studies have shown that favorable recovery from VSN was associated with increased activation in the left prefrontal and right parietal regions (17). Moreover, increasing numbers of fMRI studies have also reported that VSN might involve not only attention networks but also other brain functional networks.

According to the theory of the interhemispheric rivalry model, it is believed that a hemispheric imbalance in excitability severely affects functional recovery after stroke (4). Previous findings suggest that interhemispheric excitability is rebalanced by applying low-frequency rTMS to the contralateral hemisphere and transmitting it to the distant site through synapses (18). Consistent with our study, low-frequency (1 Hz) rTMS over the left PPC has been shown to reduce the severity of left spatial neglect (19, 20). Imaging evidence also suggests that TMS over the left PPC administered for two weeks in patients with left spatial neglect after stroke reduces the overexcitation of the frontoparietal loop (21). Furthermore, rTMS has been shown to increase activation in the fronto-parietal network and to induce a neuroplastic response leading to long-term potentiation (22). Our study evaluated dynamic neurophysiological changes bilaterally by investigating the effect of inhibiting the right PPC using rTMS. To probe the underlying mechanisms mediating the improvement of VSN, each hemisphere was analyzed separately. As expected, the P300 of the left hemisphere was increased following right PPC rTMS.

ERP is used to evaluate the effects of rTMS because it can identify the neural mechanisms underlying task-relevant SA at a finer temporal scale, thereby assessing instantaneous fluctuations. Furthermore, ERP analysis provides a more direct measure of attentional processing than behavioral data alone (23). A recent study showed not only that the P300 amplitude is reduced during early rehabilitation, but also that this reduction could serve as a predictor of negative outcomes in patients with stroke occurring in the region of the middle cerebral artery (24). Previous studies on ERP after TMS have shown that the P300 amplitude significantly increased when a single pulse was applied over the prefrontal area among healthy participants (12). In this study, patients with VSN exhibited an increase in P300 amplitude on the contralateral side of the lesion rather than on the lesion side following rTMS.

It is known that P300 is a late positive cognitive component of ERP. It is considered a useful and sensitive tool for evaluating effects on cognition as well as for examining connections to improvements in cognitive function and activation of the cerebral cortex (25). The amplitude of P300 is proportionally related to the amount of attentional resources allocated during a given task, and the associated latency is related to the speed of cognitive processing of attentional resources (26). The change in P300 amplitude in this study may be related to the cue-target paradigm, which highlights cognitive deficits in visual SA. Further, the change in P300 amplitude observed in our experiment confirmed improvement in cognition after the treatment of VSN. This paradigm comprises a series of interspersed trials, including a location cue of a different type followed by a target requiring visual spatial information processing (27); it involves contributions of executive function demands not only in attention but also in visual spatial processing (28), which reflects the allocation of neural resources regulated by cognition. Analysis of the target-induced P300 reflects the dynamic changes in brain activity related to visual SA and has a resolution of milliseconds. Previous studies focusing on auditory paradigms have reported similar performance. A meta-analysis by Jeon et al. found that the P300 elicited by auditory paradigms is relatively more influenced by genetic factors (e.g., in the case of patients with schizophrenia), while the P300 elicited by visual paradigms is more suitable for assessing symptom severity (29).

Our results suggest that inhibitory rTMS to the left hemisphere reduces RT by facilitating visual spatial processes. We found that both visual detection and shifts in attention within our paradigm increased the cognitive burden by increasing visual spatial information. This finding provides new insight for the role of visual spatial information processing in cognitive proportional improvement, though many previous studies have reported positive effects of TMS acting to produce cognitive enhancement (30). For example, participants who received a single pulse of TMS over the frontal eye field just before the onset of a stimulus exhibited enhanced performance (31). This suggests that a single pulse of TMS can increase cortical excitability for a brief period. In fact, short trains of high frequency rTMS appear to directly facilitate cortical processing; for example, Sole-Padulles et al. administered 5 Hz rTMS over the prefrontal cortex and found that this substantially enhanced the performance of face-name memory tasks in 40 participants with memory impairment (32). Additionally, functional MRI of the right prefrontal cortex and bilateral posterior cortical regions was associated with increased activity in a previous study, suggesting that rTMS could promote the recruitment of neural compensatory networks. As a further example, Snyder et al. applied TMS to a group of cognitively impaired patients in very restricted areas and found positive effects for literal and non-symbolic tasks (33). Furthermore, a study by Oliveri et al. found that 1 Hz rTMS applied over the parietal cortex increased participants' performance in a visual search task (34). Overall, the current literature suggests that TMS may enhance cognitive skills and might possibly accelerate the learning process.

This work assessed the electrophysiological processes involved in visuospatial attention to evaluate the efficacy of TMS in VSN. Although, we aimed to discover trends in order to generate hypotheses for further study. This study should be interpreted in light of its limitations. First, this study compared the electrophysiological changes post-rTMS according to time without a sham group. Therefore, a further study including a sham group will be needed in the future. Second, this was a single-center study with only 10 patients; thus, the small sample size may be a cause of bias and may affect the results. Third, there was no follow-up of the patients who completed the rTMS treatment, making it impossible to determine the persistence of the intervention effect. Further, the P300 amplitude may have been confounded by other clinical variables such as dietary and circadian factors. However, P300 is still considered as a useful tool for evaluating activation of the cerebral cortex associated with cognitive information processing. High density EEG combined with sophisticated signal processing algorithms and TMS-EEG can provide much more information about the neurophysiological characteristics and brain dynamics of cortical brain areas or networks as compared to ERP with 64 channels. Finally, we only evaluated participant responses after an effective prompt, though an unexpected target may generate more obvious responses. Given that patients did not adequately respond to invalid prompts, we were unable to perform statistical analysis on invalid prompts. Therefore, in the future, we plan to include long-term follow-up to evaluate the long-lasting benefits of rTMS and to use imaging techniques to provide theoretical support for the mechanisms underlying recovery after treatment.

In conclusion, through the electrophysiological evaluation of patients with VSN before and after TMS treatment, we provided direct evidence of the role of low-frequency rTMS in SA. Specifically, contralateral low-frequency rTMS treatment resulted in an increase in P300 amplitude (the late component of stimulating lateral attention), reflecting an improvement of cognition in VSN. It is possible that rTMS enhances cognitive ability by improving the balance between the hemispheres and plasticity of brain processing, leading to an increase in task allocation on the treatment side. This suggests that rTMS possibly acts through a compensation mechanism for tasks performed by the contralateral hemisphere, supporting the compensation theory of the healthy hemisphere. The parameters used in this study are a valuable reference for the selection of clinical VSN treatment strategies.

## Data Availability Statement

The original contributions presented in the study are included in the article/supplementary material, further inquiries can be directed to the corresponding author/s.

## Ethics Statement

The studies involving human participants were reviewed and approved by the Ethics Committee of Xuanwu Hospital (the teaching hospital affiliated with Capital Medical University; approval number [2019]016) and was conducted in accordance with the principles of the Declaration of Helsinki and its later amendments. The patients/participants provided their written informed consent to participate in this study. Written informed consent was obtained from the individual(s) for the publication of any potentially identifiable images or data included in this article.

## Author Contributions

L-lY: drafting and revising the manuscript, study concept and design, acquisition of data, analysis and interpretation of data, and obtaining funding. H-xX: analysis and interpretation of data. LC: study concept and design, revising the manuscript, and obtaining funds. W-qS: study concept and design, analysis and interpretation of data, and study supervision. All authors contributed to the article and approved the submitted version.

## Funding

This work was supported by the Beijing Hospitals Authority Youth Programme [Grant Number QML20180806] and the National Natural Science Foundation of China [Grant Number 82002386]. The funding sources had no involvement in the study design, in the collection, analysis and interpretation of data, in the writing of the report, or in the decision to submit this article for publication.

## Conflict of Interest

The authors declare that the research was conducted in the absence of any commercial or financial relationships that could be construed as a potential conflict of interest.

## Publisher's Note

All claims expressed in this article are solely those of the authors and do not necessarily represent those of their affiliated organizations, or those of the publisher, the editors and the reviewers. Any product that may be evaluated in this article, or claim that may be made by its manufacturer, is not guaranteed or endorsed by the publisher.
